# The cancer metabolic reprogramming and immune response

**DOI:** 10.1186/s12943-021-01316-8

**Published:** 2021-02-05

**Authors:** Longzheng Xia, Linda Oyang, Jinguan Lin, Shiming Tan, Yaqian Han, Nayiyuan Wu, Pin Yi, Lu Tang, Qing Pan, Shan Rao, Jiaxin Liang, Yanyan Tang, Min Su, Xia Luo, Yiqing Yang, Yingrui Shi, Hui Wang, Yujuan Zhou, Qianjin Liao

**Affiliations:** 1 Hunan Key Laboratory of Cancer Metabolism, The Affiliated Cancer Hospital of Xiangya School of Medicine, Hunan Cancer Hospital, Central South University, 283 Tongzipo Road, 410013 Changsha, Hunan China; 2 University of South China, 421001 Hengyang, Hunan China

**Keywords:** Metabolic reprogramming, Immunity, Oxysterols, TME, TIL, Immune checkpoint…

## Abstract

The overlapping metabolic reprogramming of cancer and immune cells is a putative determinant of the antitumor immune response in cancer. Increased evidence suggests that cancer metabolism not only plays a crucial role in cancer signaling for sustaining tumorigenesis and survival, but also has wider implications in the regulation of antitumor immune response through both the release of metabolites and affecting the expression of immune molecules, such as lactate, PGE_2_, arginine, etc. Actually, this energetic interplay between tumor and immune cells leads to metabolic competition in the tumor ecosystem, limiting nutrient availability and leading to microenvironmental acidosis, which hinders immune cell function. More interestingly, metabolic reprogramming is also indispensable in the process of maintaining self and body homeostasis by various types of immune cells. At present, more and more studies pointed out that immune cell would undergo metabolic reprogramming during the process of proliferation, differentiation, and execution of effector functions, which is essential to the immune response. Herein, we discuss how metabolic reprogramming of cancer cells and immune cells regulate antitumor immune response and the possible approaches to targeting metabolic pathways in the context of anticancer immunotherapy. We also describe hypothetical combination treatments between immunotherapy and metabolic intervening that could be used to better unleash the potential of anticancer therapies.

## Introduction

Metabolism involves a network of biochemical reactions that convert nutrients into small molecules called metabolites [1]. Through these conversions and the resulting metabolites, cells generate the energy, redox equivalents and macromolecules (including proteins, lipids, DNA and RNA) that they require to survive and sustain cellular functions [2]. It is well known that normal cells get energy via first glycolysis in the cytosol that is followed by mitochondrial oxidative phosphorylation (OXPHOS) under aerobic conditions. When oxygen is scarce, the cells rely on glycolysis rather than oxygen-consuming mitochondrial metabolism for energy supply. However, the metabolic pattern of tumors is different from that of normal cells [3]. As first observed by Otto Warburg, the phenomenon that cancer cells prefer to perform glycolysis in the cytosol even in the presence of oxygen, was known as ‘‘Warburg effect’’ or “aerobic glycolysis” [4]. Because the infinite proliferation of tumor cells requires faster energy supply, the ATP production rate of glycolysis is much faster than that in the oxidative phosphorylation although efficiency in ATP production per molecule of glucose is much lower via glycolysis [5, 6]. In fact, various central metabolism pathways can be dysregulated in cancer cells [2]. More importantly, emerging evidence indicates that cancer cells are able to suppress anti-tumor immune response by competing for and depleting essential nutrients or otherwise reducing the metabolic fitness of tumor-infiltrating immune cells [7, 8].

Both the innate and adaptive immune systems have now established roles in the host defense against cancers through various mechanisms, which are raising an unprecedented development of modern cancer immunotherapies. The innate immune system consists of different populations of immune cells, containing macrophages, neutrophils, monocytes, eosinophils, basophils, and natural killer cells, which are responsible for innate immunity against pathogens to maintain homeostasis of the host [9]. Indeed, immune cells are capable of sensing various signals in the microenvironment and turning on specific immune functions in response. More and more evidences have pointed out that the immune response is associated with dramatic modifications in tissue metabolism, including the depletion of nutrients, increased oxygen consumption, and the generation of reactive nitrogen and oxygen intermediates [10–12]. Similarly, many metabolites in the tumor microenvironment, in turn, also influence immune cell differentiation and effector function [13, 14]. But recent works reveal that immune cells compete with cancer cells and other proliferating cells in the microenvironment for nutrients [15]. Therefore, it suggests that metabolic interventions hold promise for improving the effectiveness of immunotherapies.

The previous studies showed that the modifications of cancer cell metabolism are, in part, due to the recruitment of many inflammatory and immune cells [16]. Subsequently, more and more researchers have found that the aberrant metabolites or intermediates of cancer metabolism could have an important role in regulating the proliferation, differentiation, activation and function of immune cells [17–19]. Recent studies found that our immune system was closely related to other metabolic functions (including cancer) in a way that has never been realized before. Moreover, therefore it is described as a new field called immunometabolism [20]. However, the actual process of how metabolic reprogramming and cancer immune response affect each other has not been understood. In this review, we will discuss this process from the following aspects.

## Main text

### Overview of metabolism of tumor cells and immune cells

#### 1. Metabolism of tumor cells

**Fig. 1:**
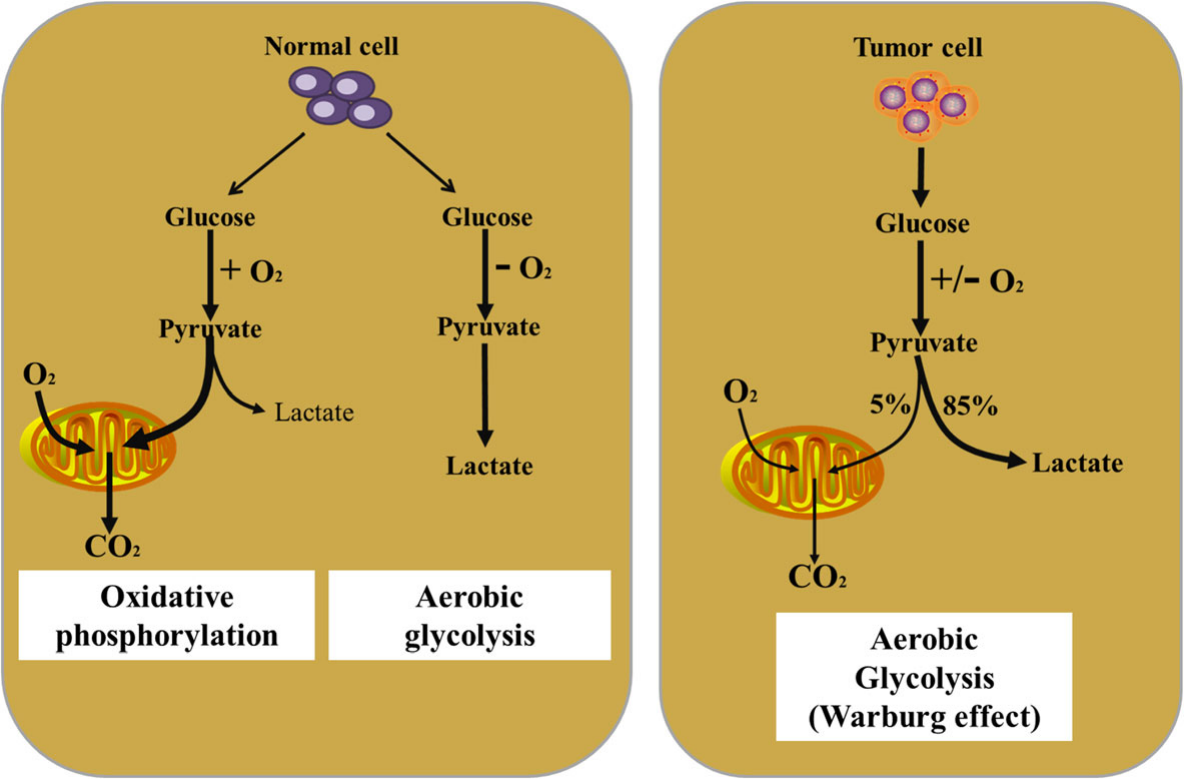
Regulation of glucose metabolism in cancer cells. Glucose metabolism mainly contains glycolysis and TCA cycle in the mitochondrion. These pathways are generally altered in tumor cells compared to normal cells

Energy metabolism reprogramming, which fuels fast cell growth and proliferation by adjustments of energy metabolism, has been considered as an emerging hallmark of cancer [4]. Because tumor is a heterogeneous disease, and its heterogeneity in cells and structure gives it a complex metabolic pattern. In fact, tumor cells mainly use the glycolysis pathway to quickly provide ATP (adenosine triphosphate) for their own growth, and also provide biological macromolecules for cell replication through the pentose phosphate pathway (PPP) and serine metabolism pathway [2, 7]. For instance, under hypoxic conditions, tumor cells usually produce pyruvate through the glycolysis pathway, which in turn produces lactic acid instead of entering mitochondria and converting to Acetyl-CoA to produce ATP [1]. However, even under sufficient oxygen conditions, tumor cells still preferentially use glycolysis to generate ATP, which is a well-known aerobic glycolysis (Warburg effect) **(**Fig. 1**)** [4]. At the same time, tumor cells will not only decompose glucose for providing ATP, but also use glutamine, serine, arginine, fatty acids and lipid substances to promote their own proliferation [21]. Interestingly, tumor cells will choose different metabolic methods to produce ATP and biological macromolecules for their own use according to the concentration of external nutrients and different stress conditions.

For example, under the stress condition of nutrients deprivation such as glucose or glutamine, tumor cells activate oncogene c-Myc to support the survival of tumor cells by regulating the expression of metabolic enzymes such as PHGDH, PSAT1, PSPH and other metabolic enzymes in the serine synthesis pathway and enhancing the de *novo* synthesis of serine and maintaining redox homeostasis [22]. In addition, under hypoxic or nutrient-deficient stress conditions, tumor cells produce acetyl-CoA by ingesting the smallest two-carbon fatty acid (e.g., acetoacetate), to provide energy for their own survival and bio-macromolecules such as fatty acids to promote their own survival [23–25]. Similarly, the metabolites produced by tumor cells through the decomposition of ketone bodies could enter the TCA (tricarboxylic acid) cycle to provide ATP for cell survival [26]. Therefore, the metabolic mode of tumor cells is complex and changeable, and it will choose the optimal metabolic mode for its own survival according to its different environment.

#### 2. Metabolism of immune cells

The immune system contains a variety of immune cells, such as macrophages, neutrophils, monocytes, eosinophil, basophils, lymphocytes and natural killer cells. These cells are at rest when the body is in a steady state, and will be quickly activated and respond when the body is stimulated by infection, inflammation or other external substances [27]. In the previous section, we briefly introduced the complex metabolic modes of tumor cells. Interestingly, these complex and varied metabolic methods also exist in immune cells [28]. The recent research shows that there is a significant difference in energy use between immune cells in the resting state and the activated state. Among many immune cells with different functions, T cells play an important role in clearing pathogens and killing cancer. T cells will show completely different metabolic patterns according to different activation states [29]. For instance, the metabolism of naïve T cell is basically static, showing zero proliferation, so only the most basic nutrient intake, minimum glycolysis rate and minimum biosynthesis need to be maintained. ATP is mainly produced by OXPHOS [30]. Once activated by an external stimulus into an effector T cell (Teff), it appears as a metabolic activation state, increasing nutrient absorption, increasing glycolysis rate, synthesis accumulation of protein, lipid and nucleotide [30]. At the same time, the mitochondrial oxygen consumption is reduced, and eventually T cells gain the ability to grow and proliferate and produce progeny cells to exert effect killing function.

Interestingly, the metabolic pattern of memory T cells is similar to that of the naïve T cells with maintaining basic nutrient intake, lower glycolysis rate and relying on OXPHOS to provide ATP [30, 31]. In addition, the activated neutrophils, M_1_ macrophages, iNOS-expressed dendritic cells (DCs) mainly rely on glycolysis for energy supply. Glycolysis plays an important role in the activation of DCs; however, DC mainly uses oxidative phosphorylation for energy metabolism in the resting state. Meanwhile, the activation of DC has also changed the lipid metabolism and affected its function. Besides, aerobic glycolysis and pentose phosphate pathways are the main metabolic modes of neutrophils. Glycolysis regulated many important functions of neutrophils, such as respiratory burst and chemo-taxis. More interestingly, after the activation of B lymphocytes caused by LPS stimulation or antigen stimulation, glycolysis and mitochondrial metabolism are enhanced. But the glycolysis is the major metabolism in activated B lymphocyte. Differently, the regulatory T cells (Treg cells), M_2_ macrophage mainly rely on OXPHOS from fatty acid oxidation (FAO) to supply energy [32]. Different metabolic patterns also affect the differentiation of different immune cell subgroups (Table 1). Therefore, exploring the metabolic reprogramming mechanism of immune cells and the effect of metabolism on immune cell functions will not only help to understand the essence of immune response and its regulatory mechanism.

**Table 1:** Different metabolic ways of immune cells

Type	Cell subtypes	Metabolic patterns	Reference
T cell	Naïve T cell	FAO OXPHOS Glutamine metabolism	[33–35]
	Treg cell	OXPHOS FAO	[36]
	Effector T cell	Glycolysis OXPHOS	[37, 38]
	Memory T cell	FAO OXPHOS	[39, 40]
B cell	B cell (resting) Activated B cell	Glycolysis Glycolysis	[41, 42]
Macrophages	M_1_	Glycolysis Pentose phosphate pathway	[43, 44]
	M_2_	OXPHOS FAO	[45, 46]
Neutrophils	-	Glycolysis	[47, 48]
NK cell	-	Glycolysis	[41]
DCs	DCs (resting)	OXPHOS	[49, 50]
	DCs (active)	Glycolysis	

## Competition for nutrients between tumor cells and immune cells

Metabolic transitions are not unique to cancer cells, but are also characteristic of other rapidly proliferating cells, such as activated T cells, Treg cell, neutrophils and so on [28]. Glucose is the nutrient that tumor cells absorb and consume the most, and is the most dependent, and it is also an important energy substance necessary for immune cell activation, differentiation, and function [51, 52]. The tumor microenvironment (TME) is accompanied by different degrees and types of immune cell infiltration. Like cancer cells, tumor-infiltrating lymphocytes (TIL) require nutrients found within the TME to support proliferation and differentiation [53]. The researchers found that tumors inhibited the function of tumor-infiltrating T cells through competitive uptake of glucose, even when there are enough tumor antigens for T cells to recognize [54]. Indeed, several recent studies demonstrated that the glycolytic activities of cancer cells may restrict glucose consumption by TIL T cells, thereby inducing T-cell exhaustion and immune escape [55]. For instance, an accelerated glucose metabolism in renal cell carcinoma (RCC) tissues is associated with a low infiltration of CD8^+^ effector T cells, as demonstrated by an inverse correlation between GLUT1 expression and infiltrating T cell numbers in a tissue microarray analysis of RCC specimens [56]. In fact, a large amount of glucose uptake by the tumor in the microenvironment is bound to inhibit T cell function by affecting T cell metabolic patterns.

**Fig. 2:**
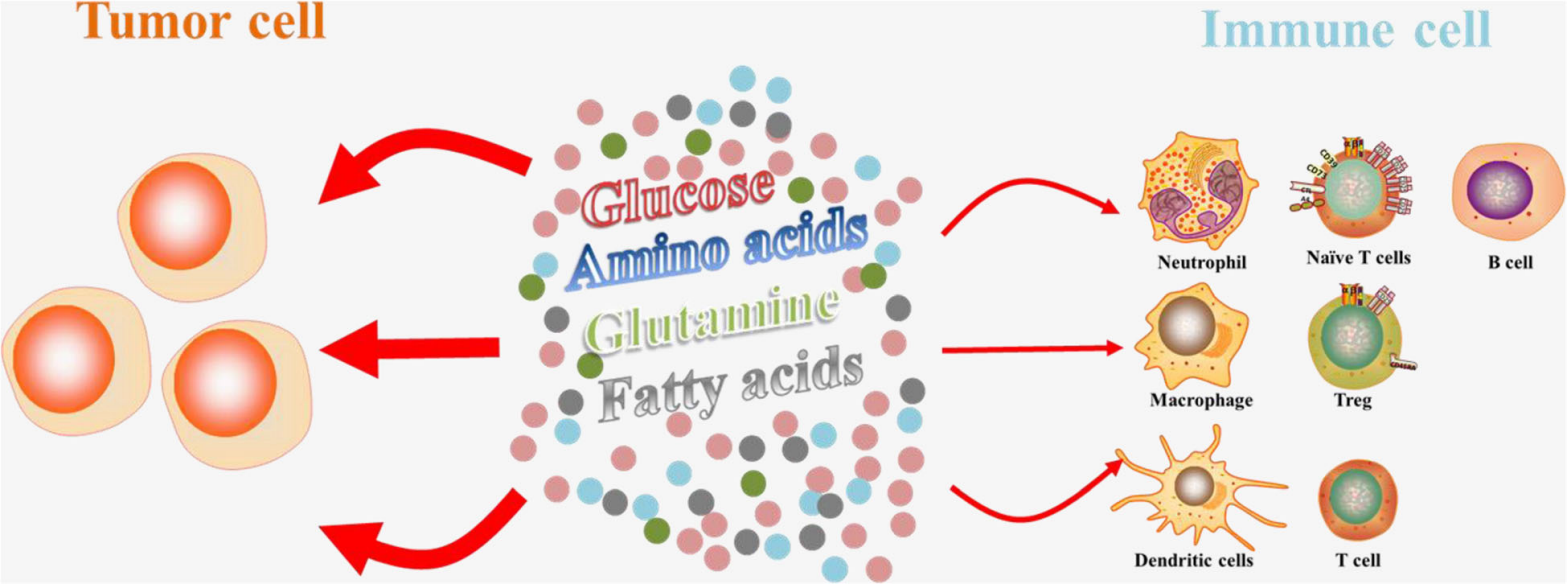
The nutritional competition between tumor cells and immune cells inside tumors. The competition-caused deficiency of glucose, glutamine, and fatty acids and a couple of amino acids are known to affect the function of immune cells, including Treg, macrophages, dendritic cell, NK cells and so on

According to the literatures reported, glycolytic metabolites also have a deleterious effect on immune function [57]. Although the competitive uptake of glucose under tumor microenvironment conditions is responsible for the damage to T cell function, the competitive uptake of amino acids, glutamine, fatty acids and other metabolites or growth factors by tumor cells and immune cells and the expression of corresponding transporters on the cell surface are still important factors affecting the functioning of immune cells (Fig. 2) [15]. Moreover, high levels of lactate and low pH, hypoxia and high levels of ROS are likewise prevalent in the TME, eventually leading to cancer progression and immune escape [57]. Thus, targeting these metabolic pathways in tumors could be a promising strategy to overcome the deleterious effects of metabolic competition between the tumor and the immune system, and enhance tumor immunogenicity [58]. However, further investigations are needed to test this hypothesis in the tumor setting both in preclinical models and in future human studies.

## Effect of tumor metabolic reprogramming on immune cells

In addition to nutrient consumption, metabolites produced by cancer cells can have a profound effect on immune cells in the microenvironment [57]. For example, lactate, a byproduct from elevated aerobic glycolysis in cancer cells, induces apoptosis via reduced expression of the autophagy factor FIP200 in naïve T cells in both patients with ovarian cancer and mouse models [59]. However, the immune cells that function in the immune system are by no means only T cells and NK cells. And metabolites produced by tumor metabolic reprogramming are complex and changeable. Although tumor cells are mainly powered by aerobic glycolysis of glucose, there are other metabolic methods, such as amino acid metabolism, glutamine metabolism, fatty acid metabolism, cholesterol metabolism, etc. We will discuss the effect of tumor metabolic reprogramming on immune cell function from the following aspects.

### 1. The effect of tumor metabolites on immune cell

#### Lactate

 The aberrant glycolysis of tumor means that tumor cells consume a lot of glucose and produce large amount of lactic acid even in the presence of sufficient oxygen, with a correspondingly low rate of OXPHOS. Lactic acid accumulates in cells and then is exported into the extracellular environment via activating monocarboxylate transporters (MCTs) on cell membrane, in particular, monocarboxylate transporter 4 (MCT4), which ultimately results in establishing an acidic TME [60]. The low pH of the TME has been shown to be beneficial for the selection of more aggressive tumor cells and suppress tumor immunity to promote tumor progression [61]. In fact, the previous and recent research both reported that both aerobic glycolysis and the resultant acidification of the TME have been shown too strongly influence T cell mediated antitumor immune responses and the activities of tumor-infiltrating myeloid cells [62]. In addition, the recent clinical studies have found that the serum lactic acid level increases significantly with the increase of the patient’s tumor burden [62]. Actually, lactate produced by tumor cells might contribute to tumorigenesis by promoting IL-23-mediated and IL-17-mediated inflammation [63]. In addition to modulating immune responses, lactic acid produced by cancer associated fibroblasts (CAFs) can be used by tumor cells as an alternative nutrient source [64].

In addition, lactic acid will also affect the function of NK cells, and then impair the secretion of IFN-γ [65, 66]. Mechanism research shows that excessive intake of pathological concentrations of lactic acid by NK cells can cause intracellular acidification and inhibit the up-regulation of the nuclear factor of activated T cells (NFAT) signal, resulting in reduced production of NFAT-regulated IFN-γ and promoting apoptosis [67, 68]. More importantly, both human and rat melanoma present the high levels of lactate. For instance, in immune-competent mice, reducing the production of lactic acid can slow the tumorigenic ability and the infiltration of CD8^+^ T cells and NK cells secreting IFN-γ in the tumor is significantly increased [67]. However, in mice lacking lymphocytes and NK cells, there was little difference in tumorigenic ability between the low lactate group and the control group. Interestingly, a recent research discovered that lactate produced by glycolysis of tumor cells in the tumor microenvironment activates mTOR pathway, thereby phosphorylating the transcription factor TFEB and inhibiting its nuclear translocation, thereby inhibiting the expression of ATP6V0d2 (vacuolar ATPase subunit) in macrophages. The inhibition of ATP6V0d2 could mediate HIF-2α lysosomal degradation and program TAMs (tumor-associated macrophages) in the tumor microenvironment into immune cells that promote tumor growth [69]. Similarly, a negative correlation between intratumoral lactate concentration and overall survival in patients with cervical cancer has been reported [68]. Together, these findings suggest that the higher lactate content in the tumor and the accompanying acidified TME will suppress immune cell function and abrogate immunosurveillance of cancer, ultimately leading to immune escape.

#### Glutamine

 Glutamine metabolism as a whole is a crucial element of cancer cell metabolism. Glutamine is important for nucleotide synthesis, amino acid production, redox balance, glycosylation, extracellular matrix production, autophagy, and epigenetics [70]. Subsequently, studies have shown that glutamine plays an important role in the growth of normal cells and cancer cells [71]. Under conditions of nutritional deficiencies, cancer cells can obtain glutamine by breaking down large molecules. For example, excessive activation of the oncogene RAS can promote endocytosis, and cancer cells clear extracellular proteins and degrade into amino acids including glutamine, providing nutrients for cancer cells [72]. In addition to tumor cells, glutamine has a high utilization rate in immune cells to support cell fate determination and immune responses, such as lymphocytes, macrophages, and neutrophils [73–75]. According to the study reported, glutamine deprivation can suppress T cell proliferation and cytokine production. However, glutamine restriction during T cell activation *in vitro* has been shown to promote memory CD8^+^ T cell differentiation [76].

In fact, glutamine metabolism plays an important role in the activation of immune cells and regulating the transformation of CD4^+^ T cells to inflammatory subtypes [74, 77]. It is now clear that the high utilization rate of glutamine in many immune cells is related to the functional activity of immune cells, such as cell proliferation, antigen presentation, synthesis and secretion of cytokines, production of NO and peroxide, phagocytosis, etc. These functions are all indirectly or directly dependent on NADPH reserves [78]. Moreover, glutamine has a high utilization rate in macrophages, and neutrophils. With the increase in utilization of glutamine, the apoptosis rate of immune cells was significantly reduced. For instance, glutamine reduces neutrophil apoptosis by reducing the expression of pro-apoptotic proteins Bax and Bcl-xs [79]. The production and secretion of pro-inflammatory cytokines (IL-6, IL-1, and TNF) through macrophages are also controlled by the obtainability of glutamine [80]. Therefore, the metabolism of glutamine plays an essential role as a modulator and synergetic supporter for the activation of a macrophage. In addition, by blocking the glutamine pathway in cancer cells to increase the content of amino acids in the tumor microenvironment and enhance the killing effect of immune cells. Jonathan D. Powell and his team found that blocking glutamine can induce different metabolic processes, thereby overcoming the immune escape of tumors [81]. It suggests that targeting the glutamine metabolism will become a novel treatment of cancer.

#### **PGE**_**2**_

 Arachidonic acid is an important class of eicosapentaenoic acid related to essential fatty acids in tumor cells, and it is an important substrate for the synthesis of prostaglandins. PGE_2_ is an important cell growth and regulation factor in the cell and it is also a kind of highly active inflammatory mediator in the inflammatory response [82] and can also participate in the immune response as an immunosuppressive factor [83]. Cancer-associated fibroblast (CAF)-derived PGE_2_ or other sources of PGE_2_ could induce cancer cell invasion and participate in tumor progression by stimulating angiogenesis, cell invasion and metastasis, and inhibits apoptosis through a variety of signal pathways to promote cell survival [84]. In addition, PGE_2_ can also affect other cells in the microenvironment through autocrine and paracrine methods, such as destroying the immune response [83]. For instance, PGE_2_ is also used as an anti-inflammatory molecule to suppress the immune response via inhibiting Th1 differentiation, B cell function, T cell activation and allergic reactions, which would enhance the anti-tumor immunity [85]. In addition, PGE_2_ can also exert anti-inflammatory effects on natural immune cells, such as neutrophils, monocytes and natural killer cells [86, 87]. More importantly, the accumulation of PGE_2_ secreted by tumor cells can transform M_1_ macrophages with tumor suppressing effect to M_2_ macrophages that promote cancer [88]. The PGE_2_ secreted by tumor cells also can stimulate bone marrow-like cells to secrete cancer-promoting CXCL1, IL-6 and granulocyte-colony-stimulating factor (G-CSF), and inhibit the secretion of TNF-α and IL-12 in myeloid cells stimulated by lipopolysaccharide (LPS), and inhibit the activation of type I interferon-dependent innate immune cells, and inhibit T cells from targeting tumor antigens, and then achieve the purpose of immune escaping and tumorigenesis [89].

According to literature reports, PGE_2_ plays an important role in lymphocyte development and proliferation, and participates in the body’s immune regulation, including inhibition of T lymphocyte proliferation, differentiation and cytokine secretion *in vitro*. High levels of PGE_2_ can not only directly regulate the malignant biological behavior of tumor cells, but also prevent the pro-inflammatory response in various immune cells and mediate the reprogramming of TME, making TME in an immunosuppressive status [90]. Studies have shown that PGE2 damages the malate-aspartate shuttle (MAS) system via activating the cAMP-PKA pathway, causing the decrease of aspartic acids and a variety of enzymes and intermediate metabolites and the growth arrest of CD8^+^ T cells [91]. Subsequently, the researchers found that PGE_2_ have shown a strong synergistic effect in inhibiting the killing function of TIL cells or regulating the secretion of TIL cell-related cytokines [92]. Some researchers have reported that PGE_2_ could increase the expression of the Treg-specific marker Foxp3 and stimulate the functions of Treg cells *in vitro* and in a mice lung cancer model [93]. More importantly, the activated Treg cells also induced COX-2 expression and PGE_2_ production, which then support immune-suppressive functions by themselves. Additionally, a previous study reported that PGE_2_ could impair the function of tumor-associated DCs (dendritic cells) [94]. In this article, the authors pointed out PGE_2_ promoted tumor evasion through activating β-catenin signaling to inhibit the function of CD103^+^ DCs in the TME. Blocking the production of PGE_2_ by tumor cells may be a good anti-tumor immunotherapy method.

#### Arginine

 Most tumor cells lack ASS1 (argininosuccinate synthetase 1), a key enzyme that produces arginine, and therefore will cause the loss of intracellular arginine synthesis capacity [95]. In this case, tumor cells will use exogenous arginine to make up for the lack of arginine caused by the lack of key metabolic enzymes in the cell. Interestingly, arginine metabolism also has crucial roles in T cell activation and modulating immune responses. For instance, supplementation of arginine stimulates T cell and NK cell cytotoxicity and effector cytokine production *in vitro* and, in combination with anti-PD-L1 antibody treatment, significantly enhances antitumor immune responses and prolongs the survival of osteosarcomabearing mice [96].

In the cell, arginine mainly produces urea, L-ornithine and nitric oxide, L-melon through the reaction of arginase (ARG) and nitric oxide synthase (NOS). Some studies have reported that the accumulation of ARG1-expressing immunomodulatory cells, including M2-like tumor-associated macrophages (TAMs), tolerogenic DCs and Treg cells, in the TME might suppress antitumor immunity by degrading arginine and thus limiting the availability of this amino acid to T cells [97]. More importantly, studies have pointed out that the arginine absorbed by tumor cells in the tumor microenvironment is mainly related to tumor provided by bone marrow cells (macrophages, monocytes, myeloid inhibitory cells, neutrophils, etc.). It means that tumor cells will consume a large amount of arginine in the TME, which will cause the lack of arginine in the TME, so the activation of antitumor immune cells is bound to be inhibited [97]. Therefore, replenishment of arginine and prevention of arginine degradation in the TME are attractive strategies to re-invigorate T cell mediated and NK cell-mediated immune responses [98]. Moreover, arginine supplementation during *in vitro* expansion of T cells promotes their differentiation to central memory-like T cells with superior antitumor activity.

#### Tryptophan

 Tryptophan is an essential amino acid necessary for organisms to carry out protein synthesis and other life metabolic activities. Actually, tryptophan degradation is mainly converted by two different dioxygenases IDO1 (indoleamine-2, 3-dioxygenase) and TDO2 (tryptophan-2, 3-dioxygenase) to tryptophan to kynurenic acid [99]. Expression of high levels of these tryptophan-degrading enzymes in tumor cells promotes tumor progression and is correlated with a worse prognosis in patients with gastric adenocarcinoma [100]. In fact, high levels of IDO and TDO in tumors have been suggested to decrease tryptophan availability in the TME, which in turn suppresses the tumoricidal functions of T cells. Because the activation of T cells is extremely sensitive to the concentration of tryptophan in the peripheral environment, the tryptophan in the microenvironment will be metabolized and utilized by tumor cells in a large amount, which will cause the lack of tryptophan and trigger the apoptosis of T cells [17, 101]. Furthermore, the accumulation of tryptophan induced by systemic IDO inhibition promotes tumor regression by increasing the production of cytokines, including IL-12 and IFN-γ, and tumor infiltration of T cells and neutrophils in mouse metastatic liver tumor and bladder tumor models. The IDO1 inhibitor has been shown to alleviate immunosuppression in the TME and promote the activation of tumor-specific T cells in preclinical models [101]. The outcomes of combined treatment with IDO1 inhibitor plus the immune checkpoints inhibitors (ICIs) are being assessed in patients with melanoma. IDO inhibition might impair NAD^+^ generation not only in immunosuppressive and/or pro-tumor TAMs but also in CD8^+^ TILs. Importantly, production of NAD^+^ is implicated as a crucial event that sustains T cell immune responses [102]. However, other IDO inhibitors are undergoing clinical testing, mostly in combination with ICIs.

#### Fatty acids

 Tumor cells often have increased rates of de *novo* fatty acid synthesis to divert energy production into anabolic pathways for the generation of plasma membrane phospholipids and signaling molecules. Meanwhile, fatty acid synthesis provides cell membranes and other key lipid cell structures necessary for immune cell proliferation [103]. The studies have reported that fatty acid synthesis is necessary for inflammatory macrophages to differentiate and function. However, some studies pointed out that effector immune cell grow rapidly and require lipids formed by fatty acid synthesis to build cell membranes during proliferation, while memory immune cells grow slowly and biosynthesis needs relatively little, so fatty acid oxidation (FAO) is the main [104]. Actually, the aberrant accumulation of lipid metabolites (e.g., short-chain fatty acid, long chain fatty acid, cholesterol, etc.) in tumor-infiltrating myeloid cells, including MDSCs, DCs and TAMs, has been shown to skew these immune cells towards immunosuppressive and anti-inflammatory phenotypes via metabolic reprogramming [103]. Nowadays, many scholars believe that the regulation mechanism of fatty acids on immune cells is not that fatty acids change the composition of fine membranes or become inflammatory mediators, but that fatty acids directly participate in signal transmission in cells.

#### Cholesterol

 Cholesterol is an important part of the surface of the cell membrane. Rapidly proliferating cells require more membrane structure and more cholesterol synthesis. High expression of cholesterol in tumor cells can protect tumor cells from immune surveillance and other treatments [105]. Emerging evidence suggests the importance of cholesterol metabolism in innate immune response. The high level of cholesterol caused by tumor cell can promote the expression of suppressive immune checkpoints of T cells, thereby making it lose its anti-tumor effect [106]. More importantly, researchers found that the cholesterol concentration in tumor cells is much higher than immune cells, and the higher the cholesterol concentration in immune cells, the higher the expression of immune detection points such as PD-1, LAG-3, and TIM-3, etc. Similarly, immune cells are also prone to apoptosis, and the lower the cytotoxicity and proliferation capacity of the cells. In fact, high cholesterol can disrupt the lipid metabolism network in T cells, thereby exerting the function of suppressing immunity [107].

Oxysterols, the metabolites originating from autoxidation or enzymatic oxidation of cholesterols, are oxygenated enzymatically (e.g., 25-hydroxycholesterol (25OHC), 27-hydroxycholesterol (27OHC) 22-hydroxycholesterol (22HC) and 24S-hydroxychlesterol (24OHC)), while some are not produced enzymatically (e.g., 7alpha/beta-hydroperoxycholesterol (7OOHC), and 7 ketocholesterol (7KC)) [108]. They are present in very low concentrations in mammalian systems, always accompanied by a high excess of cholesterols [109]. The 27OHC is elevated in both breast tissues and tumors in patients with estrogen receptor-positive breast cancer [110]. Elevating the level of 27OHC promotes cell proliferation and tumor growth by modulating a series of gene expression including estrogen-receptor signaling genes, such as ARMT1 and PARD6B, as well as genes involved in the GDFN–RET signaling pathway [11]. In addition, oxysterols also have multiple functions in shaping the immunological landscape. For instance, the accumulation of 22OHC can recruit CD11b^high^Gr1^high^ neutrophils in the conditioned medium of various cancer cells, which are emerging as an important immunosuppressive population in the TME [111]. Besides, 24OHC and 27OHC also recruit neutrophils in other cancer types. Especially, 27OHC has been found to deplete cytotoxic CD8 T cells, thereby promoting tumor metastasis [112]. Actually, cholesterols can be transformed into a variety of substances with important physiological effects in the body, e.g., adrenal cortex hormones, androgens, estrogen, progesterone, 7-dehydrocholesterol, vitamin D3, bile acids, etc. Among them, adrenal cortex hormones could suppress the function of all immune cells [113]. Other cholesterol-derived mediators have an important role in immune regulation, but different mediators have different regulating effects on immunity. However, it’s well known that decreasing the high cholesterol concentration in TME helps to relieve the immune suppression of T cells.

**Fig. 3:**
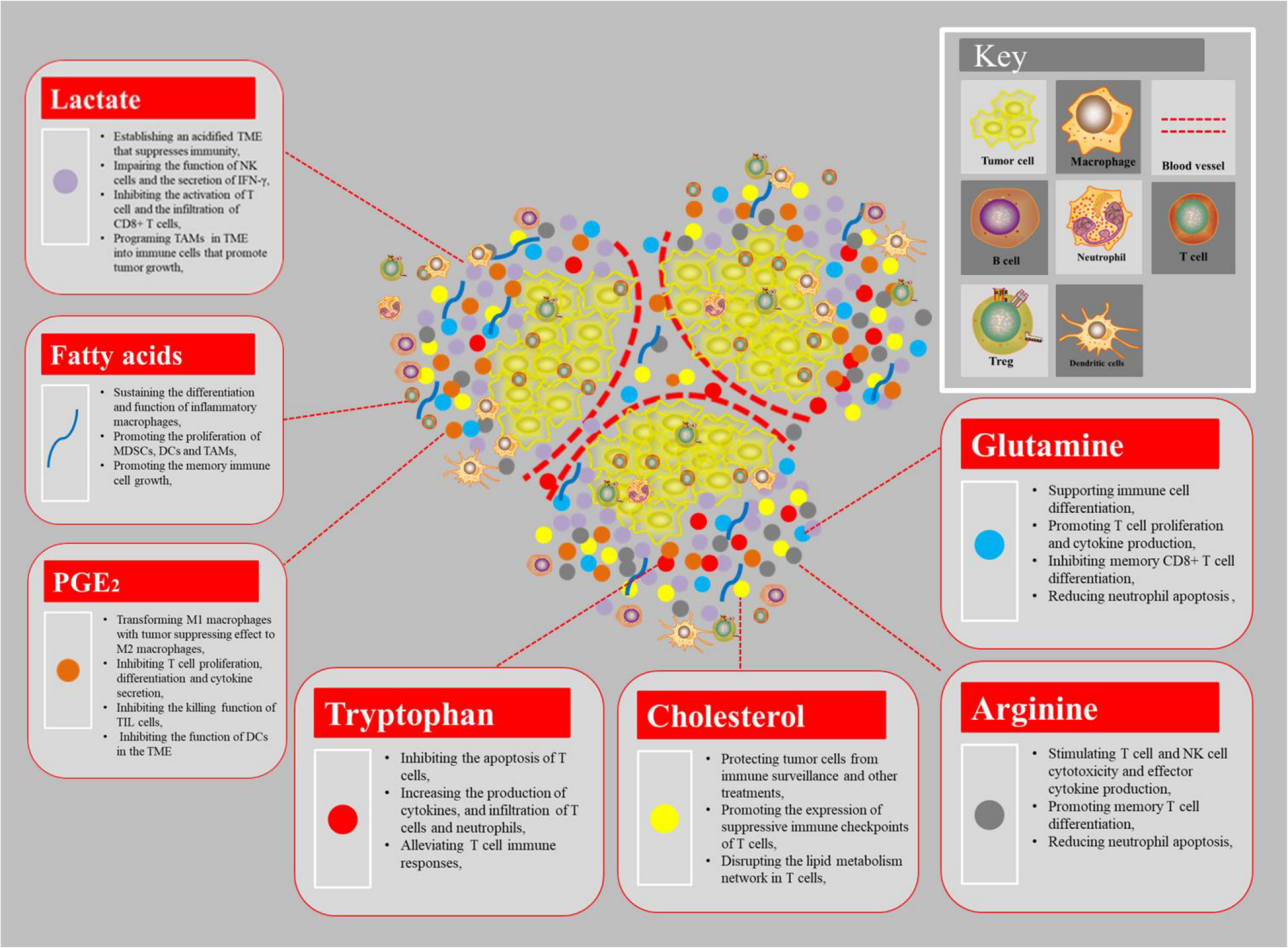
The regulation of metabolites produced by tumor cells in immune cells The metabolites are known regulators of immune cell function, such as lactate, fatty acid, PGE_2_ and arginine, etc. As a consequence, the accumulation of extracellular lactate and the amino acids arginine, tryptophan, and glutamine in the tumor environment could affect the proliferation, function and differentiation of immune cells, and the production of cytokines. The glucose-deprived, lactic acid-enriched TME impairs T cell and NK cell function and thus antitumor immune responses and polarizes tumor-associated macrophages (TAMs) towards a generally pro-tumor, M2-like phenotype. Competition for amino acids, including arginine and tryptophan, between immune cells and tumor cells can also suppress antitumor immunity. The availability and usage of fatty acids in immune cells within the TME are also influenced by competition with tumor cells. Notably, a high rate of cholesterol esterification in the tumor can impair immune responses and, therefore, disruption of cholesterol esterification in order to increase the concentration of cholesterol in the plasma membranes of immune cells might increase their proliferation and improve their effector function. The generation of prostaglandin E2 (PGE_2_), by tumor cells and other immunomodulatory cells is also implicated in the suppression of antitumor responses

Metabolites produced by cancer cells can have a profound effect on immune cells in the microenvironment. However, the products produced by tumor cell metabolism are diverse, and their functions on immune cells are also different. In the above, we focused on the impact of lactate and other metabolites on immune cells (Fig. 3), but the impact of other metabolites on immune cells still needs a lot of research.

#### 2. The effect of metabolic enzymes on tumor immunity

Metabolites are the essential substrates for epigenetic modification enzymes to write or erase the epigenetic blueprint in cells. Thus, the availability of nutrients and activity of metabolic pathways strongly influence the function of metabolic enzymes. Consistent with findings from tumor models, the results of several clinical studies revealed that aerobic glycolytic activity in human tumors is negatively associated with host antitumor immune responses and therapeutic outcomes of anticancer immunotherapy [114]. In fact, the elevated glycolysis is the metabolic basis for trained immunity to provide the energy substrates for the enhanced activation of trained innate immune cells. For example, the three rate-limiting enzymes in the glycolytic pathway, including hexokinase 2 (HK2), phosphofructokinase 1 (PFK1), and pyruvate kinases type M2 (PKM2) acted as a hallmark of liver cancer and is responsible for the regulation of immune evasion via regulating by many mechanisms, such as the AMPK, PI3K/Akt pathway, HIF-1α, c-Myc and noncoding RNAs in HCC (hepatocellular carcinoma) [115]. Besides the glycolytic enzymes, the enzymes about cholesterol metabolism and transport have important effect on immune cell function, production, and activity [116]. Researchers found that the cholesterol esterase ACAT1 in the T cell metabolism pathway is a good regulatory target. Inhibiting the activity of ACAT1 can greatly improve the anti-tumor function of CD8^+^ T cells (also known as killer T cells) [117]. Because after ACAT1 is inhibited, the free cholesterol level on the killer T cell membrane increases which makes the T cell tumor antigen immune response more efficient. In addition, the extracellular nucleotidase CD39 and CD73 can significantly up-regulate the extracellular adenosine content of tumors, and can maintain the expression of a chronic innate immune response, leading to immune tolerance, and then leading to uncontrolled tumor growth [118]. Although successive studies have pointed out the important regulatory role of metabolic enzymes in tumor immunity, its specific regulatory functions and mechanisms still need a lot of research to support it.

### 3. The effect of metabolic pathways on tumor immunity

#### mTOR pathway

**Fig. 4:**
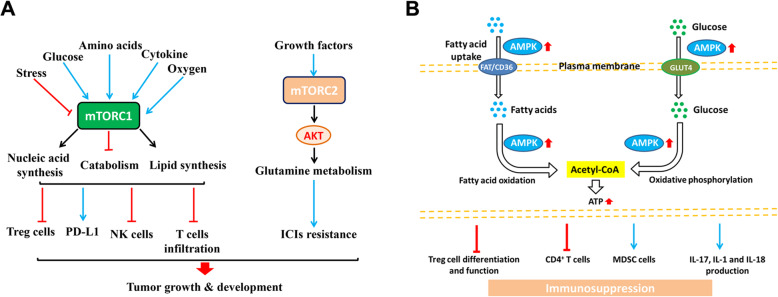
The regulation of metabolic pathway in immune cells. **a** The activation of mTORC1 induced by various factors (cytokines, glucose and oxygen, etc.) up-regulates PD-L1 expression, and inhibits the Treg cells, NK cells and T cell infiltration. In addition, the growth factors-induced mTORC2 activation could increase AKT expression and affect the glutamine metabolism, ultimately causing the ICIs (immune checkpoint inhibitors) resistance and promote the cancer growth and development. **b** AMPK activation enhances the uptake of fatty acids and glucose via FAT/CD36 and GLUT4 respectively. Meanwhile, the activation of AMPK increases the fatty acids oxidation and oxidative phosphorylation in mitochondria to elevate the intracellular ATP level. Ultimately, these alterations commonly affect the functions of immune cells, such as inhibiting Treg cells differentiation and function, decreasing CD4^+^ T cell activity, inducing MDSC cells (marrow-derived suppressor cells) and elevating the secretion of IL-17, IL-1, and IL-18, which ultimately lead to the immunosuppression

 Mammalian/mechanistic target of rapamycin (mTOR) is a serine/threonine protein kinase that is highly conserved from yeast to mammals, and it plays an important role in regulating cell growth and metabolism [119]. mTOR has two different structures and functions: mTOR complex 1 (mTORC1) and mTOR complex 2 (mTORC2). Actually, mTORC1 can promote anabolism such as protein and nucleic acid synthesis, and inhibit catabolism such as autophagy. In addition, it is notable that mTORC2 can be involved in glutamine metabolism where it can increase the uptake of glutamine by regulating its cell surface transporters through activation of AGC kinases [119]. A recent study summarized that the mTOR signaling pathway of tumor cells in the tumor microenvironment promoted the tumorigenesis and development by affecting tumor immunity (Fig. 4a) [120]. For instance, gene knockdown or the use of rapamycin inhibitors to block the mTOR signaling pathway can promote the expression of Treg cells. The study of mouse lung cancer model found that mTORC1 can promote the expression of PD-L1, so that tumor cells can avoid the killing of immune cells. Studies on melanoma have found that tumor cells secrete inhibitory cytokines, which reduces the infiltration of T cells into the tumor microenvironment, making tumor cells resistant to immune checkpoint inhibitors after activation of AKT induced by mTORC2 [119]. mTOR exerts an influence in the interaction of many kinds of cells and in many links, such as regulating the function of CD8^+^ T, thereby promoting the immune killing effect of T cells on tumor cells, and inhibiting the activation of NK cells, and finally playing an immunosuppressive effect. The mTOR pathway can directly act on the energy metabolism of tumor cells, or it can play a role by affecting immune cells [121]. In future studies, it is necessary to further clarify the mechanism of action of the mTOR pathway in different tumor cells and corresponding immune cells, and evaluate the overall therapeutic effect of mTOR inhibitors, so that it can be better used in tumor treatment.

#### AMPK Signaling

 AMPK (AMP-activated protein kinase) is a key molecule in the regulation of cell energy homeostasis. The activation of this kinase responds to the stress of cells depleting intracellular ATP, such as low glucose, hypoxia, and ischemia and heat shock. As a cell energy detector that responds to low ATP levels, AMPK actively regulates signaling pathways that replenish intracellular ATP supply, including fatty acid oxidation and autophagy [122]. AMPK negatively regulates ATP-consuming biosynthetic processes including gluconeogenesis, lipid and protein synthesis. Interestingly, AMPK activation regulates cellular immunity in cooperation with immune signaling pathways and controlling energy metabolism which consequently affects the activation of immune cells (Fig. 4b) [123]. There is convincing evidence that AMPK activation prevents inflammatory responses through the inhibition of pro-inflammatory signaling pathways. For example, in macrophages, the activation of AMPK promotes the polarization of M_1_ pro-inflammatory macrophages towards the M_2_ anti-inflammatory phenotype [124]. Moreover, AMPK activation regulates the signaling of the anti-inflammatory cytokine in macrophages, e.g., enhancing the IL-10-induced suppression of LPS-stimulated cytokine production [125]. Several studies have also demonstrated that the activation of AMPK has an important role in the differentiation and functions of T lymphocytes by regulating their energy metabolism. These observations clearly indicate that AMPK signaling controls the balance between energy metabolism and immune responses.

#### Adenosine signaling pathway

 The concentration of adenosine in tissues is markedly increased within a few hours following tissue injury, as well as in hypoxic tissues and the TME. Accumulation of the nucleoside adenosine in the tumor microenvironment has been shown to inhibit the anti-tumor function of various immune cells, including cytotoxic T cells and natural killer cells, by binding to cell surface adenosine 2A receptor (A2AR). The ectonucleotidases CD39 (also known as NTPDase 1) and CD73 (5ʹ-NT) are cell surface molecules with pivotal roles in controlling the production of adenosine through the catabolism of ATP to AMP and AMP to adenosine, respectively [126]. Treg cells can express CD39 and contribute to immunosuppression in the TME via the adenosine-A2AR signaling axis. Actually, adenosine signaling via A2AR negatively regulated the production of type 1 cytokines and enhanced the production of IL-10 by cAMP/protein kinase A and caused signal transducer and activator of transcription 5 (STAT5) dephosphorylation, which resulted in reduced IL-2R signaling in T cells and inhibition of the nuclear factor kappa B (NF-κB) pathway [127]. More importantly, targeting CD39 and CD73 activity to inhibit adenosine production is an attractive strategy for enhancing antitumor immunity. E.g., Treg co-express CD39/CD73 on the surface and generate extracellular adenosine, contributing to immunosuppressive activities, because the activation of adenosine signaling pathway mediated the suppression of Tregs by PGE_2_ receptors expressed on T cells, leading to the up-regulation of adenylate cyclase and cAMP activities [125–127]. Therefore, tumors use the adenosinergic pathway by increasing adenosine production to promote Treg activity, aiming at an immunosuppressive microenvironment to escape immune surveillance and promote cancer growth.

**Fig. 5:**
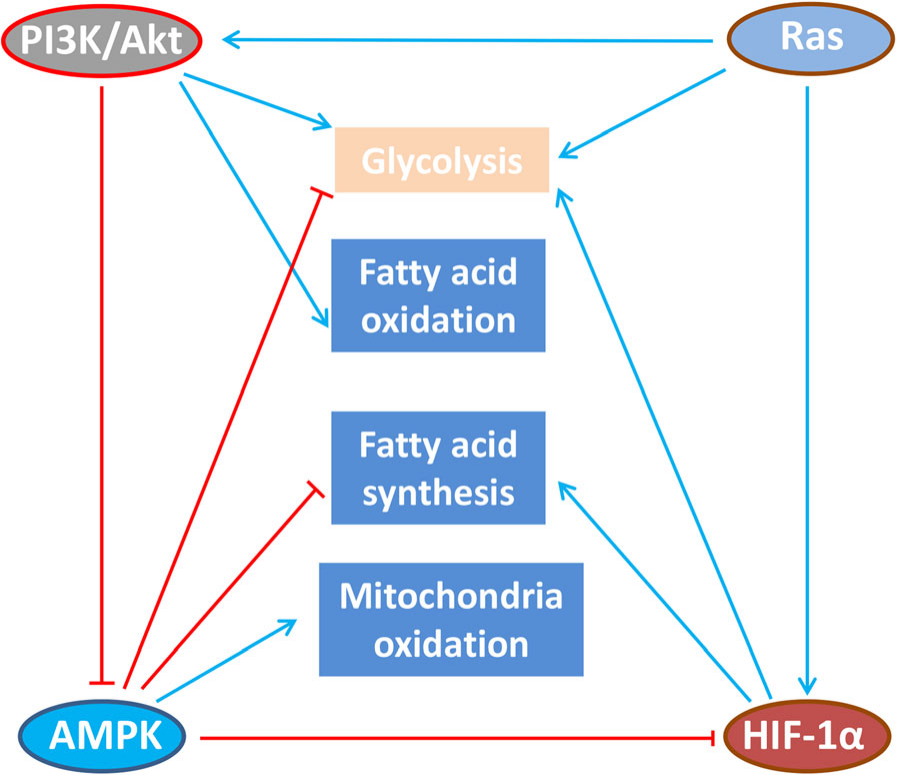
Cross-regulation of immunometabolic signaling pathways in T cell. Under hypoxia conditions, HIF-1α signaling activates anabolism-associated programs, such as glycolysis and fatty acid synthesis; however, these mechanisms require additional investigation in primary T cells. AMPK directly inhibits HIF-1α. During activation and/or replete nutrient conditions, PI3K-AKT and Ras signaling promotes glycolysis, fatty acid oxidation. PI3K-Akt can reportedly inhibit the AMPK, but this regulation is not yet reported to occur in T cells

There are several different signaling pathways involved in cell metabolism that influence the cancer T cell immunity, such as PI3K/AKT, Ras, insulin receptor, MAPK, HIF-1α signaling pathway (Fig. 5) [128, 129], etc. However, the concrete mechanism is not very clear and need further study.

## Effect of immune cell metabolic reprogramming on tumor immunity

The activated process of immune cell requires a large amount of energy and metabolic intermediates to meet the needs of biosynthesis, so as to complete the proliferation, differentiation and execution of effector functions. Its metabolic pattern is completely different from that of inactivated immune cells, which is very similar to the growth of tumor cells. The phenomenon of “metabolic reprogramming” has occurred [28]. At the same time, the phenotype and function of immune cells will be regulated by metabolism. Therefore, different metabolic pathways regulate the growth, differentiation and function of immune cells [2]. Among them, the research on glucose metabolism is the most in-depth. We will discuss the effect of immune cell metabolic reprogramming on cancer immunity from the following aspects.

### **1. Effect of immune cell metabolic pattern on immune cell functions**.

.

The known metabolic patterns of immune cells can be divided into the following three categories: (1) the activated immune cells are metabolized in a manner similar to the Warburg effect without obvious oxidative phosphorylation, (2) glycolysis in effector T cells, (3) tricarboxylic acid cycle and oxidative phosphorylation in resting immune cell. Interestingly, some studies indicated that fatty acid oxidation is also active in resting immune cell [32, 128, 129]. On one hand, immune cells in different activation states or differentiation stages exhibit different metabolic patterns. This active selection of metabolic pathways enables immune cells to adapt to their functional requirements. On another hand, the environment and metabolic state of organisms will affect the phenotype and function of immune cells (Table 2).

**Table 2:**
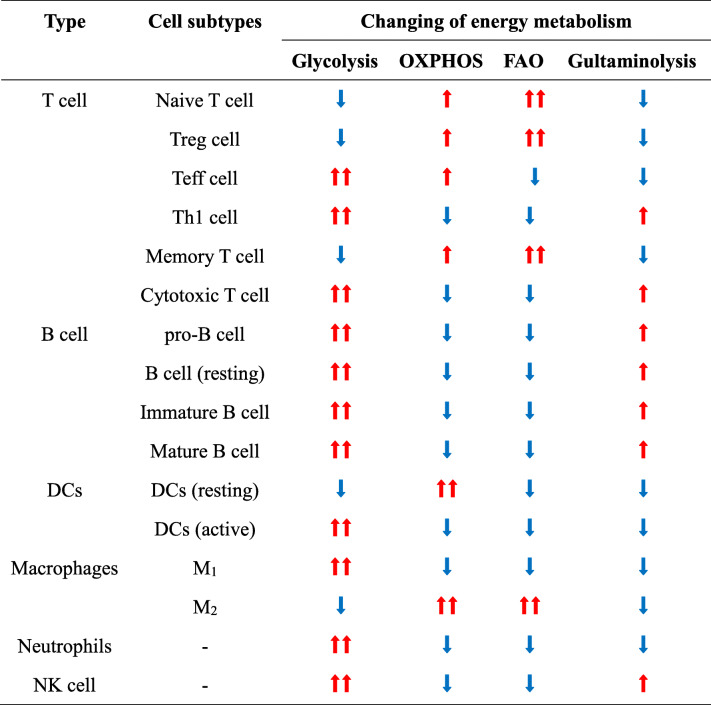
Metabolic changing of immune cells

: Significantly up-regulated; : Up-regulated; : Down-regulated

#### Glycolysis

 Glycolysis is the major metabolic pathway in neutrophils, M_1_ macrophage, dendritic cell, naïve T cell and effector T cell [30], etc. For instance, CD8^+^ T cells are a key component of adaptive immune response and play an important role in anti-tumor immunity. T cell antigen receptor (TCR) connection and subsequent co-stimulation trigger substantial changes in cell metabolism and induce the activation and proliferation of naïve CD8^+^ T cells. Although oxidative phosphorylation (OXPHOS) is the main energy source for naïve and memory CD8^+^ T cells, elevated glycolytic metabolism is a prerequisite for cell growth and expansion of the activated CD8^+^ T cell population [130]. Besides the naïve T cell and effector T cell, when the macrophages are polarized, the glucose metabolism pattern changes. For example, when M1-type stimulating factor IFN-γ and LPS co-stimulate macrophages derived from mouse bone marrow, cellular glycolysis is enhanced. More importantly, glycolysis can regulate the inflammatory response associated with macrophages [131]. Under the stimulation of LPS, the metabolic mode of mouse macrophages switches to glycolysis, resulting in the accumulation of succinate, an intermediate product of the tricarboxylic acid cycle, which can be further activated AKT-mTOR-HIF1α signaling pathway induces the expression of inflammatory factor IL-1β [132]. Glycolysis regulates many important functions of neutrophils, activated NK cell and DC cells, therefore, people expect to control the tumor immunity by regulating the glycolytic changes of immune cells, which is also an important direction for future research [133]. However, this is far from actual clinical application and need further study.

#### OXPHOS

 Regulatory T cells (Treg cells), M_2_ macrophages and memory T cells mainly rely on FAO (fatty acid oxidation)-sourced OXPHOS for energy supply. There is clear data from several groups that perturbing the OXPHOS of immune cell can inhibit its functions [134]. For instance, resting NK cells depend mainly on OXPHOS for their survival. When it’s activated by some cytokines, such as IL-2, IL-12, and IL-15, both murine and human NK cells up-regulate OXPHOS to support IFN-γ production. Interestingly, some studies confirmed that OXPHOS inhibition showed little effect on NK cell cytotoxicity. Additionally, resting T cells also mainly depend on OXPHOS, and antigen presentation and the induction of the glycolytic pathway in a mitochondria-independent manner [135]. A recent study indicated that mitochondrial OXPHOS directs lineage commitment of pathogenic Th17 cells and suppresses Treg cells by orchestrating distinct cellular and molecular events [136]. In this article, the authors pointed that it is possible that Treg cells generated with OXPHOS inhibition under Th17 conditions may exhibit distinct functional and transcriptional characteristics from classical Treg cells. That suggested that manipulating cellular metabolism, and specifically mitochondrial OXPHOS, may provide a new promising therapeutic intervention for modulating the balance between pathogenic Th17 and Treg cells in autoimmune response.

#### FAO

 It’s reported that the FAO is not the major metabolic pathway in many immune cells, such as memory T cell and activated DC cell [137]. However, the effect of FAO in regulating the function of immune cell cannot be ignored. E.g., M_2_-like macrophages are dependent on FAO to fuel their bioenergetics demands to maintain the antitumor effect; however, whether inhibition of FAO improves the antitumor activity of macrophages has not been established [138]. Moreover, the FAO plays an essential role in the generation and maintenance of Tm (memory T cell) [139]. Researchers have confirmed that FAO is the metabolic energy basis for Tm to respond to antigen stimulation in a timely manner, and is conducive to Tm mitochondria to maintain normal functions and long-term cell survival. Importantly, FAO is also very important for T cells, which can regulate the balance between Teff (effector T cell) and regulatory T cells (Treg) [140]. FAO inhibits Teff cell activation and up-regulates the expression of the inhibitory programmed death 1 receptor and CPT1a, then weakening the secretion of IFN-γ. Conversely, the expression of genes involved in FAO (including CPT1a) in Treg cells is up-regulated, and the level of FAO increases, which promotes the generation of Treg cells [140]. Tolerant cells such as M2 macrophages or Treg cells mostly live in tissue microenvironments where nutrients are relatively lacking, so the efficiency of ATP production is crucial, so it is more important to produce more ATP through FAO and maintain normal mitochondrial function. FAO plays a key role in regulating the innate and adaptive immune response, which mainly depends on the different primary needs of different immune cells. Therefore, exploring the metabolism of different immune cells is essential for a comprehensive analysis of FAO’s immune regulation.

#### Pentose phosphate pathway

 Glucose utilization through the pentose phosphate pathway (PPP) is required for several neutrophil functions [141]. In resting neutrophils, the amount of glucose metabolized via this route is only 2 to 3 percent of the total glucose consumed by the cells. The PPP is of particular importance for neutrophils because it provides the NADPH needed for *de novo* fatty acid synthesis and for NOX activity. NOX uses NADPH to reduce oxygen and generate superoxide (O_2_) [141, 142]. M_1_ type macrophages transport a large amount of glucose from outside the cell and are enriched in the cell in the form of glycogen. When glycogen is decomposed, a large amount of glucose 6-phosphate flows to the pentose phosphate pathway, which increases the ratio of NADPH/NADP^+^ and promotes the production of GSH [143]. Blocking glycogen decomposition or inhibiting the oxidation stage of the pentose phosphate pathway will cause M_1_ macrophages to reduce the GSH/GSSG ratio, increase ROS, and increase M_1_ macrophages apoptosis [144, 145]. Therefore, interfering with the flow of glycogen to PPP can promote the apoptosis of M1 macrophages mediated by ROS.

However, the roles of immune cells metabolic reprogramming in modulating function of immune cells remain to be explored. In the above, we only briefly introduced the most important metabolic changes of immune cells, but the role of other metabolic methods is still unclear, such as amino acid metabolism, glutamine metabolism, cholesterol metabolism, etc. Therefore, research on the metabolic reprogramming of immune cells needs to be strengthened.

### 2. Effect of immune cell metabolic reprogramming on immune checkpoints

Emerging evidence indicates that ICIs (immune checkpoint inhibitors) also affect the metabolic fitness of T cells. A study indicates that PD-1 and CTLA-4 receptors decrease glucose uptake, inhibit glycolysis and impair T cell activation, whereas only PD-1 engagement promotes FAO and enhances lipolysis [146]. Interestingly, PD-1 blockade reverses glucose restriction in TILs (tumor killer cells), enhancing CD8^+^ T cells glucose influx and glycolysis via mTOR signaling, which allows IFN-γ production, improving their effector anti-tumor function [8]. It suggests that the immune checkpoints are closely associated with the function of immune cells. However, it is of great interest to define whether metabolic alterations of immune cell could affect immune checkpoints. Since the metabolism of different types of immune cells is different, changing the metabolic mode of immune cells is bound to affect the expression of immune checkpoints. For example, naïve T cells are quiescent, and generate ATP mainly through oxidation of pyruvate in the TCA cycle, OXPHOS and FAO [138]. After encountering antigens that are recognized by the T cell receptor (TCR) and simultaneously activate co-stimulatory signals such as CD28, naïve T cells undergo extensive proliferation, growth and differentiation into effector T cells which are dependent on the glycolysis [52]. It’s well-known that the effector T cells usually highly express the co-stimulatory immune checkpoints and down-regulate the inhibitory immune checkpoints. The increasing of effector T cells with high expression of co-stimulatory immune checkpoints could significantly promote the anti-tumor response.

For another example, NK cells mainly dependent on OXPHOS to obtain energy in the resting state, and glycolysis increases after activation [147]. The balance between inhibitory KIRs (iKIRs) and activating KIRs (aKIRs) is essential to the function of NK cells, the inhibitory immune checkpoints on NK cells are activated so that NK cell activation is inhibited in the tumor microenvironment [148]. The activated NK cells can inhibit the iKIRs signaling pathway after activation to enhance the anti-tumor immunity via shifting from OXPHOS to glycolysis [149]. In addition, glycolytic metabolism is characteristic for active and efficient T lymphocyte, but in TME there are low levels of glucose. Besides glucose deprivation, TILs have reduced enolase-1 activity, and this deficit can be overridden if downstream pyruvate can be supplied. In melanoma a co-stimulatory TNF receptor family member 4-1BB was found elevated on TILs with CD8^+^PD-1^+^Tim3^+^LAG3^+^ phenotype [150]. Therefore, analyzing both immune checkpoints and the metabolic outline of immune cells can offer innovative insights in new therapy targets and cancer therapeutically approaches. In addition to already approved immune checkpoint inhibitors therapy in cancer, approaching metabolic points could improve therapy efficacy and hinder resistance to therapy.

### 3. Effect of immune cell metabolites on its function

**Fig. 6:**
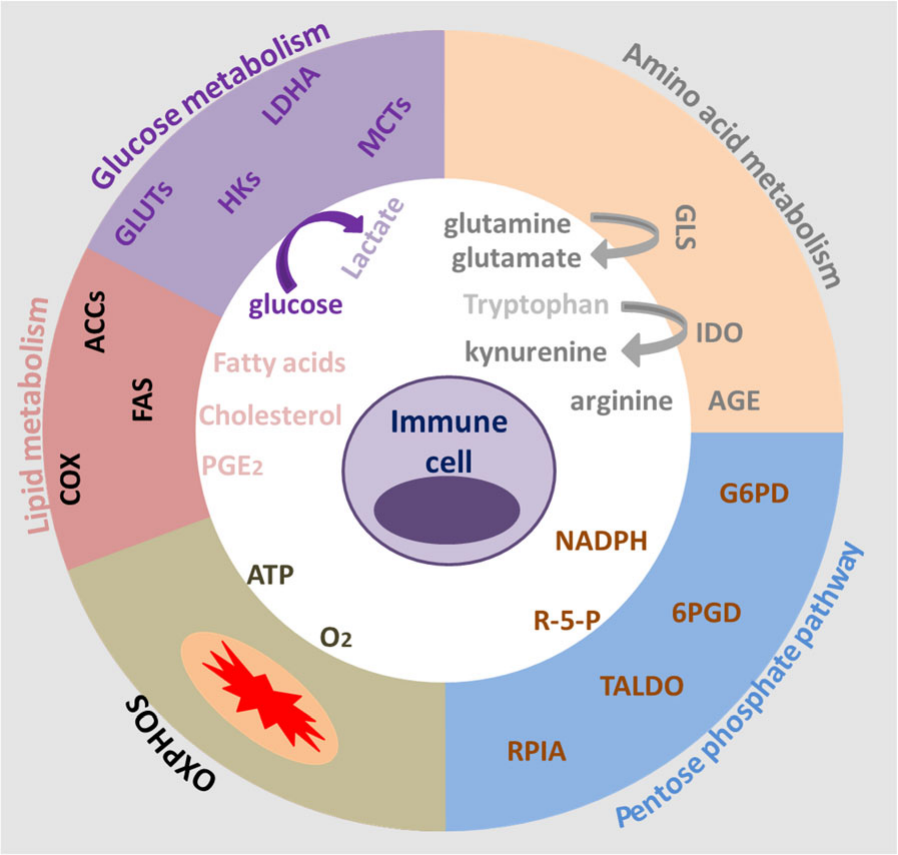
Metabolic hallmarks of immune cells and the interplay between immune cells and immune cells. Immune cells exhibit high expression of glucose transporteres (GLUT), lactate dehydrogenase (LDH), cyclooxygenase (COX), arginase (ARG), indolamine 2,3-dioxygenase (IDO), glutaminase (GLS), and oxidative phosphorylation (OXPHOS), etc. As a consequence, glucose and the amino acids arginine, tryptophan, and glutamine are depleted from the immune microenvironment and nutrient restriction leads to an anergic status of anti-tumoral cytotoxic T cells. In addition, accelerated glycolysis by some immune cells results in lactate production and secretion via monocarboxylate-transporters (MCTs). Lactate and other metabolites, such as glutamate, prostaglandins (PGE_2_), kynurenines, cholesterol and R-5-P, affect immune cells

The immune system needs a large amount of energy and metabolic intermediates to meet the needs of biosynthesis, so as to complete the execution of proliferation, differentiation and effector functions *in vivo*. The metabolic profile of immune cells is completely different from those in the resting state, and the changed metabolic intermediates and metabolic profile will further regulate the phenotype and function of immune cells (Fig. 6). Therefore, the metabolites of immune cells how to affect the function of immune cells has gradually become a research fever in immunology in recent years. We will discuss the effect of immune cell metabolites on the function of immune cell in the following aspects.

#### Glucose

 Glucose is one of important energy source for immune cells, which use glucose to produce ATP and metabolic intermediates [52]. In addition to glucose, other raw materials can also be used by lymphocytes, such as glutamine, ketone bodies and fatty acids, but glucose is still the most important nutrient for oocytescytes [52]. Recently, the team of Dr. Wanjun Chen discovered that excessive intake of glucose would affect immune cells and cause the collapse of the immune system. It turned out that excessive glucose promoted the mitochondria in T cells to produce more reactive oxygen species, and these reactive oxygen species activated some cytokines related to helper T cell Th17: TGF-β and RORγt, leading to the excessive differentiation and stimulation of Th17 cells and provoking inflammation in the body [151]. When there is a lack of glucose in the tumor microenvironment, the function of most immune cells will be defective. Low glucose levels activate the “energy receptor” AMPK kinase in the cell, which phosphorylates the mTORC1 receptor, thereby inhibiting the activity of the mTORC1 signaling pathway [152]. At the same time, low glucose levels will reduce the activity level of Hif-1α through mTORC1. More interestingly, Hif-1α itself has an inhibitory effect on the immune activity of dendritic cells [152]. Experiments show that when HIF-1α is knocked out, the synthesis of some pro-inflammatory factors (including IL12a and TNF, etc.) and immune activating factors (including CD80 and CD86, etc.) in dendritic cells will be significantly increased, which activates T cell proliferation. Importantly, T cells compete with dendritic cells for glucose uptake, causing the latter to lack glucose supply [153]. Under the action of the above mechanism, the immune activation effect of dendritic cells on T cells will become more powerful in the tumor microenvironment.

#### ATP

 Immune cells use various energy sources to produce ATP to maintain their functions. As mentioned above, intracellular ATP is to ensure cell survival and proliferation and important energy source for metabolic function [154]. However, in response to conditions such as hypoxia, apoptosis, necrosis or inflammation, ATP can release from the cell into the extracellular microenvironment in lymphocytes [155, 156]. Extracellular ATP also has an impact on specific immune responses. Studies have found that extracellular ATP can activate T cells expressing P2 × 7, inhibit the differentiation and function of regulatory T (Treg) cells, and induce them to differentiate into TH17 cells and promote the production of interleukin-17 (IL-17) in white blood cells [157, 158]. Meanwhile, ATP can also promote the activation of NLRP3 inflammasome in phagocytes, thereby releasing a large amount of IL-1 and IL-18, further aggravating the occurrence of inflammation [159]. In fact, extracellular ATP also has a regulatory effect on the functions of CD4^+^ T cells and Treg cells, and this regulation is concentration-dependent [160]. At physiological concentrations, ATP has no significant effect on the functions of the above two types of T cells, while high concentration of extracellular ATP has a bimodal effect on CD4^+^ T cell activity, and the concentration of 250 nM stimulates cell proliferation, cytokine releasing and adhesion molecule expression, thereby enhancing the immune effect, and when the concentration is 1 mM, it induces apoptosis and inhibits the activity of CD4^+^ T cells, thereby producing immunosuppressive effects [161]. Additionally, high concentration of extracellular ATP can lead to the production of high concentration of adenosine, and the adenosine produced by ATP degradation has obvious immunosuppressive effect [162]. In the future, it is necessary to design large-scale studies to determine the significance of extracellular ATP in mediating tumor immunity and its value in the treatment and prognosis of tumor patients.

#### Fatty acids

 Lipid metabolites include phospholipids, fatty acids and cholesterol, among which the research on the effects of fatty acid metabolism on immune cells is particularly extensive. The accumulation of fatty acids in monocytes with high expression of enzymes about fatty acid anabolism can promote the differentiation of monocytes into macrophages [163]. If the synthesis of fatty acids is inhibited in macrophages, the macrophages will have a more monocyte-like morphology [164]. The increase of intracellular oleic acid, a monounsaturated Omega-9 fatty acid, can induce macrophages to produce IL-1a, which can lead to inflammation [165]. Recent reports have shown that increased levels of unsaturated fatty acids (including oleic acid, linoleic acid and arachidonic acid) in cells can stimulate the production of interleukin-1α (IL-1α) in macrophages, leading to increased inflammation. More importantly, some researches pointed that palmitic acid, oleic acid, and very low-density lipoproteins (VLDL) can all enhance the endotoxin sensitivity of macrophages to varying degrees, and this phenomenon may be related to the fact that these lipids can induce NF-κB activation of macrophages [146]. Additionally, the oxidation of fatty acids also plays a role in maintaining the balance between effector T cells and regulatory T cells effect. In regulatory T cells, fatty acid oxidation is mainly used to provide energy, while in effector T cells, fatty acid oxidation is inhibited [166]. Whether or not there is such a possibility that fatty acids in effector T cells can be transferred outside the cell and taken up by Treg cells, thereby causing Treg function to be enhanced, it need further study to verify.

#### Amino acids

 Amino acids are one of the basic structural substances that constitute the body’s immune system. Amino acids are not only involved in the development of immune organs and the proliferation and differentiation of immune cells, but also affect the secretion of cytokines and the regulation of immune responses [167]. A lack of amino acids can cause atrophy of immune organs and dysfunction of immune cells [167, 168]. Reasonable supplementation of amino acids has a positive effect on regulating the immune function of the body. Glutamine, a neutral amino acid, plays an important role in lymphocyte secretion, proliferation and function maintenance [169, 170]. When lymphocytes proliferate and differentiate in large quantities after being stimulated by antigens, glutamine is not only an important precursor for nucleotide synthesis, but also an important energy source for lymphocytes [171]. The high utilization of glutamine is essential for maintaining and regulating the immune function of macrophages [172]. Besides the glutamine, the sulfur-containing amino acids such as methionine, cysteine and cystine have important immune regulation functions [173–175]. Among them, methionine can be converted into homocysteine, and the increase in the concentration of the latter can up-regulate the adhesion of T cells, monocytes and endothelial cells. Moreover, sulfur-containing amino acids can form many derivatives, the most representative of which is glutathione. Glutathione has a reducing effect on the peroxide and reactive oxygen produced by various phagocytes such as neutrophils and macrophages in the process of exerting their effects, and plays an important role in preventing the destruction of cells by peroxides [176, 177]. Reasonable supplementation of amino acids with immune regulation functions plays an important role in maintaining a moderate immune response.

## Metabolic interventions in tumor immunity

A better understanding of the processes inducing metabolic interventions in cancer cell and immune cell might also reveal new therapeutic targets. For example, pharmacological intervention has also been shown to determine metabolic fitness and persistence of the T cell [178]. Likewise, treatment of T cells with a PI3K inhibitor in vitro resulted in less differentiated cells with improved *in vivo* persistence and antitumor activity in mice, which is associated with the roles of AKT-mTOR signaling in promoting a terminally differentiated effector phenotype and increasing glycolytic flux upon T cell activation [179]. Whereas inclusion of a CD28 domain stimulates T cell glycolysis and effector differentiation, use of a 4-1BB co-stimulatory domain induces mitochondrial biogenesis, OXPHOS and subsequent memory T cell differentiation, thus resulting in better *in vivo* persistence [180]. These data indicated that the intervening metabolic patterns of T cell were associated with the function of T cell in antitumor response.

An early indication of the crucial role of metabolic modulation in activated T cells during a primary immune response came from the observation that mTORC1 inhibition with rapamycin leads to the generation of increased numbers of memory T cells after viral clearance [181]. The inhibition of mTORC2-AKT signaling or glycolysis (the metabolic signature of effector CD8^+^ T cells) during in vitro expansion of CD8^+^ T cells can also endow the cells with a memory phenotype and increased antitumor activity [182]. However, inhibition of mTOR signaling by rapamycin can inhibit T lymphocyte activation and differentiation, suggesting that rapamycin has a strong immunosuppressive function [183]. Besides, the rapamycin can inhibit cancer immune function by inhibiting CD8^+^ T cells, promoting the production of regulatory T cells and regulating dendritic cells [184]. In summary,, it is necessary to further clarify the mechanisms of action of the mTOR pathway in different tumor cells and the corresponding tumor microenvironment, and evaluate the overall therapeutic effects of mTOR inhibitors in the future, so that it can be better applied into tumor treatment.

Interestingly, not only naïve but also tumor-reactive TILs can be metabolically manipulated with AKT inhibitors during in vitro expansion, resulting in a memory-like phenotype and increased antitumor activity upon allogeneic transplantation into immune-deficient, multiple myeloma-bearing mice [178, 181]. Actually, inhibition of mTOR or the glycolytic pathway also favors T cell differentiation towards naïve and memory phenotypes, although with a dramatic reductive effect on cell proliferation [185–187]. Furthermore, enhancement of metabolic signature using a PPARα agonist enables TILs to maintain efficient antitumor activity in the TME despite being deprived of oxygen and glucose [188]. It suggests that pharmacological activation or inactivation of metabolism could enhance immune cell metabolic fitness, survival and antitumor activity.

## Conclusions

Evidently, targeting of cancer and/or immune cell metabolism can synergize with antitumor immunity. Understanding and harnessing metabolic crosstalk in tumor cells and immune cells has the potential to increase the often low response rates achieved with immunotherapies. Although various combinations of metabolic agents and immunotherapies are already applied in clinical trials, efforts to better understand the metabolic mechanisms of tumor immune evasion and the metabolic demands of immune cells are essential to fully exploiting the therapeutic potential of combination therapies. Notably, not only metabolic programming of tumor cell also influence antigen presentation and recognition of immune cell, but the metabolic programming of immune cell could affect its function, ultimately leading to the alteration of tumor immunity. Thus, metabolic interventions might not only improve immune cell responses against highly immunogenetic cancers but also increase the immunogenicity of cancer cells, thereby broadening the spectra of cancers that can be effectively treated with immunotherapy.

## Data Availability

Not applicable.

## Abbreviations

OXPHOS: Mitochondrial oxidative phosphorylation
ATP: Adenosine triphosphate
PPP: Pentose phosphate pathway
TCA cycle: Tricarboxylic acid cycle
DCs: Dendritic cells
Teff: Effector T cell
Treg: Regulatory T cells
FAO: Fatty acid oxidation
TIL: Tumor-infiltrating lymphocytes
TME: Tumor microenvironment
AMPK: AMP-activated protein kinase
Tm: Memory T cell
ARG: Arginase
NOS: Nitric oxide synthase
HK2: Hexokinase 2
PFK1: Phosphofructokinase 1
PKM2: Pyruvate kinases type M2
ICIs: Immune checkpoint inhibitors
TCR: T cell receptor
IL-17: Interleukin-17
